# Next-Generation Sequencing-Based Pre-Implantation Genetic Testing for Aneuploidy (PGT-A): First Report from Saudi Arabia

**DOI:** 10.3390/genes12040461

**Published:** 2021-03-24

**Authors:** Yusra Alyafee, Qamre Alam, Abeer Al Tuwaijri, Muhammad Umair, Shahad Haddad, Meshael Alharbi, Hayat Alrabiah, Maha Al-Ghuraibi, Sahar Al-Showaier, Majid Alfadhel

**Affiliations:** 1 King Abdullah International Medical Research Center (KAIMRC), Medical Genomics Research Department, King Saud Bin Abdulaziz University for Health Sciences, King Abdulaziz Medical City, Ministry of National Guard Health Affairs (MNG-HA), Riyadh 11426, Saudi Arabia; ahmedyu@ngha.med.sa (Y.A.); alamqa@ngha.med.sa (Q.A.); altuwaijriab@NGHA.MED.SA (A.A.T.); Umairmu@ngha.med.sa (M.U.); haddadsh@ngha.med.sa (S.H.); harbim7@ngha.med.sa (M.A.); 2 Reproductive Endocrinology and Infertility Unit, King Abdulaziz Medical City, Ministry of National Guard Health Affairs (MNG-HA), Riyadh 11426, Saudi Arabia; RabieaaH@NGHA.MED.SA (H.A.); GhuraibiM@ngha.med.sa (M.A.-G.); sa7ar222@hotmail.com (S.A.-S.); 3 Genetics and Precision Medicine Department (GPM), King Abdullah Specialized Children’s Hospital, King Saud Bin Abdulaziz University for Health Sciences, King Abdulaziz Medical City, Ministry of National Guard Health Affairs (MNG-HA), Riyadh 11426, Saudi Arabia

**Keywords:** aneuploidy, embryos, euploidy, preimplantation genetic testing for aneuploidy (PGTA)

## Abstract

Recently, high-throughput next-generation sequencing (NGS)-based preimplantation genetic testing for aneuploidies techniques came into use. This technique is essential for successful embryo transfer and accomplishing pregnancy, thus reducing the time and cost of additional cycles. In this study, we describe our first experience in introducing an NGS-based preimplantation genetic testing for aneuploidy (PGT-A) service using next-generation sequencing in King Abdulaziz Medical City located in Riyadh, Saudi Arabia. Our main goal was to report the successful implementation of this new technology in clinical practice and highlight the factors that may affect the results. In total, 200 blastomere biopsies were obtained from 36 in vitro fertilization (IVF) cycles from Saudi couples suffering from prolonged infertility or recurrent embryo transfer failure. NGS-based PGT-A was performed in all embryos. The results were analyzed in five age groups, showing that aneuploidy rates increased with maternal age. Moreover, the results also showed that complex abnormal embryos with (2–5) aneuploidy are the most common type of embryos. Additionally, our data showed that chromosome 16-related abnormality was the most frequent abnormality detected among all reported abnormalities. In conclusion, our study suggests that NGS-based PGT-A is an applicable and reliable technique for routine-based embryo screening, especially for couples suffering from recurrent miscarriages or multiple embryo transfer failures.

## 1. Introduction

Congenital abnormalities and chromosomal abnormalities in the fetus are considered one of the most important causes of infant abnormality or death [1]. One type of chromosomal abnormality is aneuploidy, which is defined as the gain or loss of one or more chromosomes from the normal chromosome number of 46 [2]. Down syndrome or trisomy 21 (T21) is one of the most common chromosomal abnormalities, occurring with a frequency of 1 per 800 live births [3], with a prevalence of 6.6 per 10,000 children in Saudi Arabia [4]. Trisomy 18 (T18), also known as Edward syndrome, and trisomy 13 (T13), also known as Patau syndrome, are amongst the most prevalent autosomal aneuploidies, with an incidence of 1 per 5000 live births for Trisomy 18 and 1 per 16,000 live births for Trisomy 13, respectively [5,6]. Furthermore, 6.7% of recurrent pregnancy loss is caused by chromosomal abnormalities in Saudi Arabia [7].

It is well established that a high incidence of chromosomal aneuploidy in human embryos, as well as oocytes, contributes to low implantation and pregnancy rates. These aneuploidies mostly occur due to errors in chromosome segregation during female meiosis and less often during consecutive embryo mitosis [8,9]; male meiosis is rarely a cause of embryonic aneuploidies. Therefore, it is crucial to address this problem by testing embryos prior to implantation into the mother’s uterus [10].

Most of the embryo selection methods that have been used previously were based on the detailed morphology of embryos. It has been demonstrated that the optimal morphological characteristics could correlate to higher implantation rates; however, morphology is a weak predictor of implantation rate and ploidy [11,12] The most common technique, still in use today for preimplantation genetic testing for aneuploidy (PGT-A), is microscopic morphology analysis [13]. However, there are many developmental abnormalities that do not affect embryo morphology. Additionally, about 50% of good morphology embryos can be aneuploid at the blastocyst stage [14]. Therefore, embryo biopsy is required for more accurate aneuploidy detection.

Despite all the controversy about which biopsy approach is more feasible or better for implantation [15,16], the current procedures for biopsy are either by cleavage-stage biopsy at day three or by trophectoderm biopsy at day five or six of the blastocyst stage. Furthermore, the embryo mosaicism phenomenon, which occurs in an early stage of human embryo development, should be also taken into consideration for more accurate aneuploidy detection [17].

There are several techniques used to determine chromosomal aneuploidy in human in vitro fertilization (IVF) embryos, one of which is fluorescence in situ hybridization (FISH). Previously, this was the gold standard technique to perform pre-implantation genetic screening, currently known as preimplantation genetic testing for aneuploidies (PGT-A) [13]. Fluorescence probes are employed to detect selected chromosomes and estimate their numbers. While it is easy to perform, its limitation lies in the ambiguity and difficulty of the interpretation of results, thus resulting in a high rate of false-positive and false-negative results. Another caveat is the inability to check more than five chromosomes in parallel [11].

In recent years, more advanced molecular tools such as array comparative genomic hybridization (CGH), single-nucleotide polymorphism (SNP) array, quantitative real-time polymerase chain reaction (qPCR), and next-generation sequencing (NGS) have been developed [10]. Until recently, qPCR and array-based technologies (SNP array and array CGH) were considered the most viable tools for PGT-A [18]. However, with a significant reduction in cost, NGS has become the technique of choice because of the additional data obtained.

PGT-A by NGS quantifies the number of chromosomes in each embryo biopsy to differentiate between chromosomally normal (euploid) embryos with 46 chromosomes and chromosomally abnormal (aneuploid) embryos [11,12]. This technique offers many advantages, perhaps the most important of which is the decreased risk of miscarriage [13]. Second, NGS-based PGT-A has a high detection rate (98%) of genetic abnormalities [19]. Thirdly, it can increase the pregnancy rate [19,20]. Finally, it reduces the number of IVF cycles needed to achieve pregnancy, potentially reducing the time and cost of extra cycles, as reported by a recent study where it decreased the overall cost by 10% [8,9,11].

Moreover, several studies have shown the feasibility of the same application by using embryo cell-free DNA from spent culture media without the need to perform invasive biopsies for the embryos [21]. However, more validation and optimization are needed for this approach to be implemented clinically.

In this study, we describe our experience in establishing a PGT-A service in King Abdulaziz Medical City located in Riyadh, Saudi Arabia. Our main goal was to report our experience of the implementation of this new technology in clinical practice. Our second goal was to investigate and explore factors that may contribute to the aneuploidy rates of the embryos. In conclusion, to the best of our knowledge this is the first study highlighting the experience of the integration of NGS-based PGT-A in a clinical setting in Saudi Arabia.

## 2. Material and Methods

### Study Approval and Consent

This study was approved by the IRB with approval number RC19/115/R of King Abdullah International Medical Research Centre (KAIMRC), Riyadh, Saudi Arabia. Participants went through a full clinical assessment at Reproductive Endocrinology and Infertility Unit National Guard Hospital (NGH), Riyadh, Saudi Arabia. Written informed consent forms for conducting the procedure for this study and dissemination of clinical data were signed by all the families included in this study.

## 3. Study Subjects

Inclusion criteria were Saudi couples suffering from prolonged infertility (>4 years), with/without advanced maternal age (>36 completed years), or recurrent embryo transfer failure (≥10 embryos in multiple transfers) with no history of previous pregnancy outcome by regular IVF cycle from October 2019 to October 2020. Exclusion criteria included women with contraindications for IVF/ICSI, Body mass index (BMI) >30 kg/m^2^, poor quality embryos not suitable for biopsy, and finally low total antral follicle count (<7 follicles).

### 3.1. Genomic DNA Extraction from Blastomere Biopsy

Blastomeres biopsies at day 3 (cleavage stage) were performed on 200 embryos obtained from participants after 36 IVF cycles. Genomic DNA extraction experiments were performed on all biopsies, including quality control samples (confirmed euploid and aneuploid biopsies) using the Ion Single Seq™ Kit (Part No. A28955, Hilden, Germany) according to the manufacturer’s protocol. The aspirated 1–2 cells were transferred to a PCR tube containing 2.5 µL 1× PBS. A total of 2.5 µL of cell extraction buffer and 5 µL of extraction enzyme master mix were added to each sample (10 µL total volume). PCR tubes were spun at 1000× *g* for 30 s to collect liquid at the bottom of the tube [22,23]. Finally, the standard thermal cycler conditions used were 75 °C for 10 min, followed by 95 °C for 4 min and holding at 22 °C [22,23]. The PCR was performed in a Veriti™ 96-Well Thermal Cycler (Applied Biosystem ^TM^, Foster City, CA, USA, Cat. No# 4375786).

### 3.2. Pre-Amplification of Genomic DNA

A pre-amplification genomic DNA experiment was performed using an Ion Single Seq™ Kit (Part No. A28955, Hilden, Germany) according to the manufacturer’s protocol. A total of 5 µL of pre-amplification enzyme master mix was added to each sample. The PCR tubes were centrifuged at 1000× *g* for 30 s to collect liquid at the bottom of the tube. The PCR tubes were kept in a thermal cycler [24]. Finally, the standard thermal cycler conditions used were 95 °C for 2 min (1 Cycle), followed by 95 °C for 15 s, 15 °C for 50 s, 25 °C for 40 s, 35 °C for 30 s, 65 °C for 40 s, and 75 °C for 40 s (12 cycle) [25]. The PCR was performed in Veriti™ 96-Well Thermal Cycler (Applied Biosystem^TM^, Foster City, CA, USA, Cat. No# 4375786).

### 3.3. Amplification of DNA Libraries

Amplification libraries were performed using an Ion Single Seq™ Kit (Part No. A28955, Hilden, Germany) according to the manufacturer’s protocol. A total of 30 µL of amplification master mix (amplification buffer, amplification enzyme, and nuclease-free water) was added to each sample. After that, 5 µL of the Ion Single Seq™ barcode adapter was added to each sample tube (total volume 50 µL). Each barcode adapter is single-use only. Then, we mixed the samples by pipetting up and down, using a new tip for each sample, and centrifuging them at 1000× *g* for 30 s to collect liquid at the bottom of the wells. Finally, the standard thermal cycler conditions used were 95 °C for 3 min (1 Cycle) followed by 95 °C for 20 s, 50 °C for 25 s, and 72 °C for 40 s (4 Cycles). After that, 95 °C for 20 s, 72 °C for 55 s (12 cycle) and holding at 4 °C [26,27]. The PCR was performed in the Veriti™ 96-Well Thermal Cycler (Applied Biosystem^TM^, Foster City, CA, USA, Cat. No# 4375786).

### 3.4. Pool, Purify, and Quantification of the DNA Libraries

A total of 40 µL of the DNA library pool was transferred to a 0.2 mL PCR tube to each sample. The tubes were heated to 72 °C for 2 min and held at 22 °C. After that, the tubes were briefly spined to collect the samples at the bottom of the tube and then transferred to the heated DNA library pool to a 1.5 mL LoBind Eppendorf tube. To each tube, 40 µL of AMP pure XP beads were added to purify the DNA libraries. The remaining step of purification was performed using the kit’s protocol. Finally, quantification DNA libraries were obtained using the protocol of Qubit ds DNA HS assay kit (Invitrogen™, Waltham, MA, USA, Cat. No# Q32851).

### 3.5. Loading of DNA Libraries Pool into Ion Chef™ Equipment

The Ion Chef™ reagent was kept outside at room temperature for 45 min before the loading of the libraries. Other consumables and solution cartridges were placed at an appropriate position into the Ion Chef™ equipment (Thermo Fisher Scientific, Waltham, MA, USA, Cat. No# 4484177) [27]. The samples were run into batches of 16 (using 510™). Finally, 4 µL of the 1 nM pooled library was transferred to position A of the reagent cartridge, and 46 µL of NFW was added to give a final concentration of 80 pM [24]. The complete workflow from sample processing to reporting was completed in 12–16 h depending on the number of samples taken simultaneously [25].

### 3.6. Initialization and Sequencing Run into Ion S5™

The Ion S5™ ExT Sequencing Reagents cartridge was kept for 45 min to equilibrate the reagent at room temperature. After that, Sequencing Reagents cartridge, Ion S5™ ExT Wash Solution bottle, and Ion S5™ Cleaning Solution bottle were kept at an appropriate position in the Ion S5™ instrument (Thermo Fisher Scientific, Waltham, MA, USA, Cat. No# A27212). Finally, the Ion 510™ chip (Cat. No # A34292) was used to perform initialization. After the completion of initialization (~50 min), the instrument was ready for a sequencing run [28,29].

### 3.7. Bioinformatics Analysis and Interpretation of NGS Data

**Figure 1:**
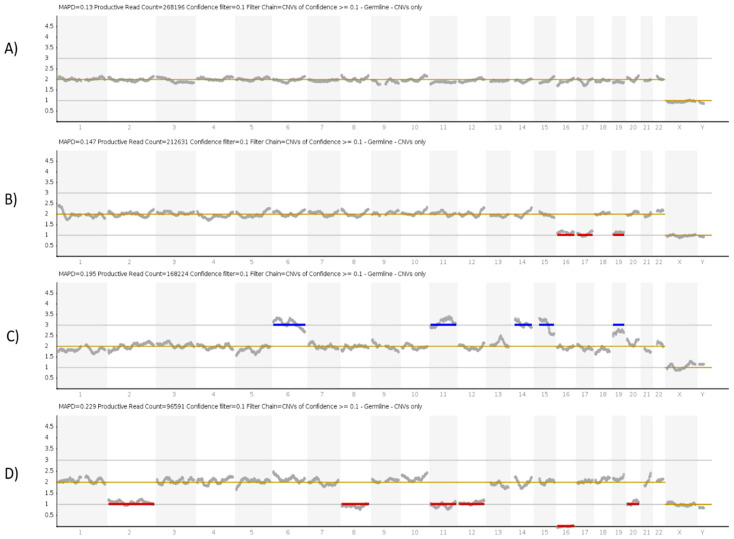
Images of ion reporter genomic viewer for different embryos. (**A**) Result of a euploid male embryo. (**B**) Result of complex aneuploidy embryo depicting three monosomies at chromosome number 16, 17, and 19. (**C**) Result of complex aneuploidy embryo depicting five trisomies at chromosome number 6, 11, 14, 15, and 19. (**D**) Result of chaotic (more than 5 aneuploidies) depicting aneuploidies at chromosomes number 2, 8, 11, 12, 16, and 20.

For data analysis, the sequencing data acquired by the IonS5™ sequencer were processed and transferred to Ion Reporter™ software. This software uses the bioinformatics tool ReproSeq w1.1 work flow to detect 24-chromosome aneuploidies from a single whole-genome sample with a low coverage [30]. Normalization was performed using the bioinformatics baseline ReproSeq Low-Coverage Whole-Genome Baseline generated from multiple normal samples. The Analysis Visualization screen opens to the IRGV Table A copy number histogram for each selected analysis appears, along with ploidy maps for selected chromosomes or chromosome regions, and karyograms showing copy number gains and losses [11,29,30]. For the purpose of this study, embryos were categorized into seven groups: euploid (no gain or loss), single-chromosome aneuploidy (only one chromosome involved), complex aneuploidy embryos (2–5 chromosomes involved either gain or loss or both), chaotic aneuploidy embryos (>5 chromosomes involved), and finally poor quality and non-informative embryos (failure of amplification), as seen in Figure 1 for a representative image of the results.

## 4. Results

In our study, blastomere biopsies from 200 embryos obtained from 36 IVF cycles (average 5.6 embryos/couple) were recruited for PGT-A using high-throughput DNA sequencing-based Ion S5 technology. Almost all of the participants were of Saudi ethnicity. The average age of all the women who participated in IVF was 35.15 years with a range of 25 to 47 years, while the average age of male participants was 44.6 ranging between 33 and 67 years. All of the general information about the participants and biopsies is summarized in Table 1.

Out of 200 samples, 46 (23%) embryos were found to be euploid and hence recommended for transfer into the respective mother’s uterus with an overall pregnancy rate of 17%. On the other hand, 120 (60%) embryos were found to be aneuploid and hence, these embryos were not recommended for transfer into the mother’s uterus. Moreover, out of 120 aneuploid embryos, we found 57 (28.5%) complex, 33 (16.5%) chaotic, 20 (10%) single-chromosome aneuploidy, and finally 7 (3.5%) partial chromosome abnormal embryos. In addition, we found 34 (17%) poor-quality embryos; these embryos were not recommended for transfer but were recommended for re-biopsy if possible. Various types of chromosomal aneuploidies/aneuploidies are summarized in Table 2.

Furthermore, the number of euploidies and aneuploidies in different maternal age groups were also examined and divided into four groups viz 25–30, 31–35, 36–40, and ≥40 years. In the first age group of 25–30 years, out of 37 embryos, 9 (24.3%) were euploid while 21 (56.8%) were aneuploid. In the second group 31–35 years, out of 59 embryos, 19 (32.2%) were euploid while 35 (59.3%) were aneuploid. In the third group of 36–40 years, out of 69 embryos, 17 (24.6%) were euploid while 42 (60.9%) were aneuploid. Finally, in the last age group, which is more than 40 years, out of 35 embryos, 8 (22.9%) were euploid while 22 (62.9%) were aneuploid. In comparison, increased age in the paternal side did not show any influence related to the total number of euploidy and aneuploidy. All the frequencies of euploidy/aneuploidy in IVF embryos of women participants within different age groups are summarized in Table 3.

Moreover, we also analyzed our data based on different types of aneuploidies such as monosomy, trisomy, complex, and chaotic in different age groups of women. We found 11 (9.2%) trisomy, 9 (7.5%) monosomy, 7 (5.8%) partial deletion, 57 (47.5%) complex, and 33 (27.5%) chaotic in the above-mentioned age groups. All these different types of chromosomal aneuploidies in different age groups are summarized in Table 4.

**Figure 2:**
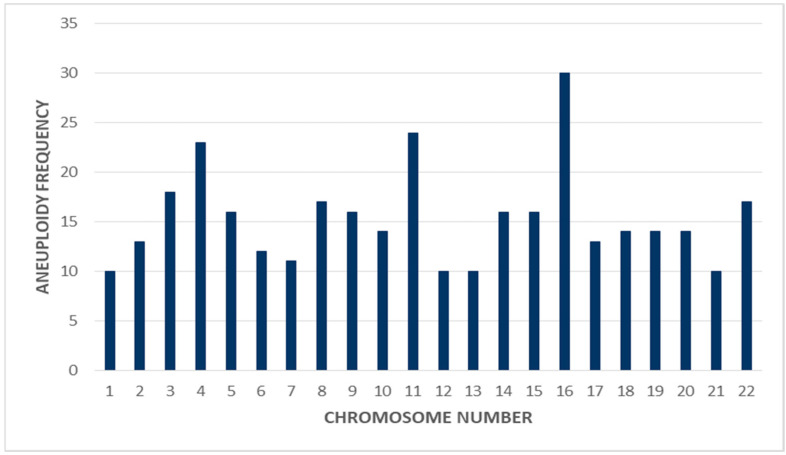
Frequency of aneuploidy for all autosomal chromosomes.

Finally, we analyzed the prevalence of aneuploid frequency in various autosomal chromosomes (1–22 pairs). The results showed that aneuploidies of chromosome 16 (30%), 11 (24%), and 4 (22%) reflect much more frequent aneuploidies than any others, as shown in Figure 2.

## 5. Discussion

In the current study, the NGS-based PGT-A technique was performed for 200 IVF embryos included in this study. It is well known that a high incidence of chromosomal aneuploidies in the human oocyte/embryo occurs in women over the age of 35 years, which consequently contributes to miscarriage and hence a low pregnancy rate after IVF implantation [11].

Therefore, maternal age is a major concern for aneuploidy and genetic disorders in the offspring in the context of the rise of IVF in mothers of increasingly older ages [12]. The association of a mother’s age and chromosomal aneuploidy in a fetus is also reflected in our study. Among the 69 IVF samples investigated in the age group of 36–40 years, only 17 (24.6%) were euploid (normal), while 42 (60.9%) showed aneuploidy (abnormal).

Furthermore, out of 35 embryos, 8 (22.9%) were euploid and 22 (62.9%) were aneuploid in the mothers whose age group was more than 40 years. In addition, complex and chaotic types of chromosomal aneuploidies were also reflected in the age group of more than 35 years. The rate of aneuploidy increased in the group of mothers over 31 years compared to the group of mothers aged less than 31 years, confirming previous trends where aneuploidy increases as the age of the mother increases [26]. In addition, in this study, we did not detect any significant influence of the paternal age on the number of euploid and aneuploid embryos. However, we cannot exclude bias related to maternal age [31].

Moreover, our result showed that the highest frequency of chromosomal abnormalities involved chromosome 16, as it appeared in 25% of all the aneuploid embryos. This finding is in accordance with previously reported data about the relation of chromosome 16 aneuploidies with frequent miscarriage and the involvement of maternal meiosis errors in the process [30]. Although not all euploid embryos transferred to their corresponding mothers yet the overall pregnancy rate was found to be 17%. Moreover, one mosaic embryo was also transferred to the mother upon the couple’s request and after detailed proper counseling, as many previous studies found that mosaic embryo can result in a healthy normal baby [32].

Consequently, the major advantages of testing embryos of those patients before implantation into the mother’s uterus are clear. Despite the various techniques that have been used previously, this study has shown that the NGS-based PGT-A platform could be a gold-standard technique for successful IVF technology. This technique has been shown to increase the chances of implantation in our study, and enabled genetically normal embryos to be transferred, thus avoiding the health complications associated with twin or triplet pregnancies.

## 6. Conclusions

This study showed the feasibility of NGS-based PGT-A in detecting chromosomal aneuploidy in the embryos. This helped the couples in our study to understand the reasons for their related infertility conditions. Moreover, it demonstrated the ability to transfer chromosomally normal embryos to increase their chances to achieve pregnancy. However, more time will be needed in order to overcome the mosaicism limitation of PGT-A that greatly affects the detection rate. To the best of our knowledge, this is the first study highlighting the experience of NGS-based PGT-A for embryo screening in a clinical care setting in Saudi Arabia. Its implication for practice could avoid the limitations of current methods and greatly benefit Saudi mothers struggling with pregnancy.

## Figures and Tables

**Table 1:** Next-generation sequencing (NGS) results of 36 preimplantation genetic testing for aneuploid(PGT-A) cycles performed in day 3 biopsies.

Results		Percentage %	Standard Deviation
No of the embryos analyzed	200	100	
Mean female age	35.15	-	4.64
Mean male age	44.5	-	8.95
Informative embryos	166	83	
Euploid embryos	46	23	
Aneuploid embryos	120	60	

**Table 2:** General PGT-A outcomes of all the embryos recruited in this study.

Result		Number of Embryos	Percentage of Total	Recommendation for Transfer	Pregnancy Rate
Euoploidy		46	23	YES	17%
Aneuploidy	Complex Abnormal Embryo	57	28.5	NO	
	Chaotic Abnormal Embryo	33	16.5	NO	
	Single Chromosome Aneuploidy	20	10	NO	
	Partial Chromosome Abnormality	7	3.5	NO	
	Mosaic Embryo	3	1.5	NO	
Poor quality embryo		34	17	NO	

**Table 3:** Frequency of euploidy/aneuploidy in in vitro fertilization (IVF) embryos of maternal and paternal participants within different age groups.

Gender	Age (Years)	No. of Embryos	No. Euploidy	% of Euploidy	No. of Aneuploidy	% of Aneuploidy	No. of Poor Quality	% of Poor Quality
Maternal	25–30	37	9	24.3	21	56.8	7	18.9
	31–35	59	19	32.2	35	59.3	5	8.5
	36–40	69	17	24.6	42	60.9	10	14.5
	≥40	35	8	22.9	22	62.9	5	14.3
Paternal	36–40	78	10	12.5	50	64.1	18	23.1
	41–45	58	8	13.8	44	75.8	6	10.3
	46–50	12	2	16.6	8	66.7	2	16.6
	≥50	62	14	22.6	34	54.8	14	22.6

**Table 4:** Types of aneuploidies found in the IVF embryos of recruited women of different age groups.

Age (Years)	T	M	PD	Mo	Complex	Chaotic
25–30	2	2	2	1	9	5
31–35	5	2	1	-	17	10
36–40	3	5	4	2	14	14
≥40	1	-	-	-	17	4
Total	11	9	7	3	57	33
% of Total	9.2	7.5	5.8	2.5	47.5	27.5

T: Trisomy; M: Monosomy; PD: Partial Deletion; Mo: Mosaic Complex: 2 to 5 aneuploidies in the same embryo; Chaotic: More than 5 aneuploidies in the same embryo. No significant differences were found between different age groups and different types of abnormalities.

## Data Availability

The supporting data on the findings of this study will be provided by the corresponding author on reasonable request.

## Abbreviations

PGT-A	Pre-Implantation Genetic Testing for Aneuploidy
PGS	Pre-Implantation Genetic Screening
PGD	Pre-Implantation Genetic Diagnosis
IVF	In vitro Fertilization
PCR	Polymerase Chain Reaction
NSG	Next-Generation sequencing
FISH	Fluorescence in situ hybridization
NFW	Nuclease Free Water
IRB	Institution Review Board
NGHA	National Guard Health Affairs
KAIMRC	King Abdullah International Medical Research Center
